# Late Intraocular Lens Dislocation Resulting From Haptic Breakage Following Cardiopulmonary Resuscitation

**DOI:** 10.7759/cureus.10456

**Published:** 2020-09-14

**Authors:** Samantha Prabakaran, Mouwafak Moureiden, Saima Qureshi, Aman Sharma, Trisha Tran, Saad Shaikh

**Affiliations:** 1 Ophthalmology, University of Central Florida College of Medicine, Orlando, USA; 2 College of Medicine, University of Central Florida, Orlando, USA; 3 Ophthalmology, Orlando VA Medical Center, Orlando, USA; 4 Ophthalmology, Howard University College of Medicine, Washington DC, USA; 5 Ophthalmology, Orlando Veterans Affairs Medical Center, Orlando, USA; 6 Ophthalmology, University of Texas Medical Branch, Galveston, USA; 7 Ophthalmology, Florida State University College of Medicine, Tallahassee, USA

**Keywords:** lens, dislocated, iol, cataract, cataract surgery, chest compressions, cardiac, haptic, haptic breakage

## Abstract

We report a case of an asymptomatic 65-year-old male who on routine eye examination had anterior dislocation of an intraocular lens (IOL) implant placed 23 years prior. Ten months prior to presentation, the patient had cardiac surgery complicated by cardiac arrest requiring chest compressions. Dislocation of an intraocular lens is a rare complication of cataract surgery. One of the causative factors for haptic breakage in our case was the polyimide haptic material. Polyimide has been shown to become brittle over time in warm and moist environments such as the human eye. This case demonstrates a case of late IOL dislocation chest compressions and, to the best of our knowledge, the first such case reported in the literature.

## Introduction

Dislocation of an intraocular lens (IOL) is a rare complication of cataract surgery which can be seen after trauma or after improper IOL fixation within the capsular bag [1,2]. Tearing of the posterior capsule, rupture of the equatorial capsule, and zonular weakness are believed to be causes of late IOL dislocation that occur beyond three months after cataract surgery [3]. IOL dislocation typically results in visual changes, such as decreased visual acuity and image distortion. It may also be associated with an inflammatory response. A less commonly reported cause of IOL dislocation is haptic breakage. We present in the following a 65-year-old male who sustained an anteriorly dislocated intraocular lens secondary to haptic breakage after undergoing chest compressions. To the best of our knowledge, this is the first such case reported in the literature.

## Case presentation

A 65-year-old male was referred to the ophthalmology department by his optometrist after identification of an anteriorly dislocated posterior chamber IOL. The patient denied vision changes or other ocular symptoms. His past ocular history was significant for uncomplicated bilateral cataract surgery in 1993, twenty-three years prior to presentation. He denied any recent ocular or head trauma. Ten months prior to presentation, the patient underwent coronary artery bypass graft surgery which was complicated by postoperative cardiac arrest for which he required cardiopulmonary resuscitation, which included chest compressions. 

**Figure 1:**
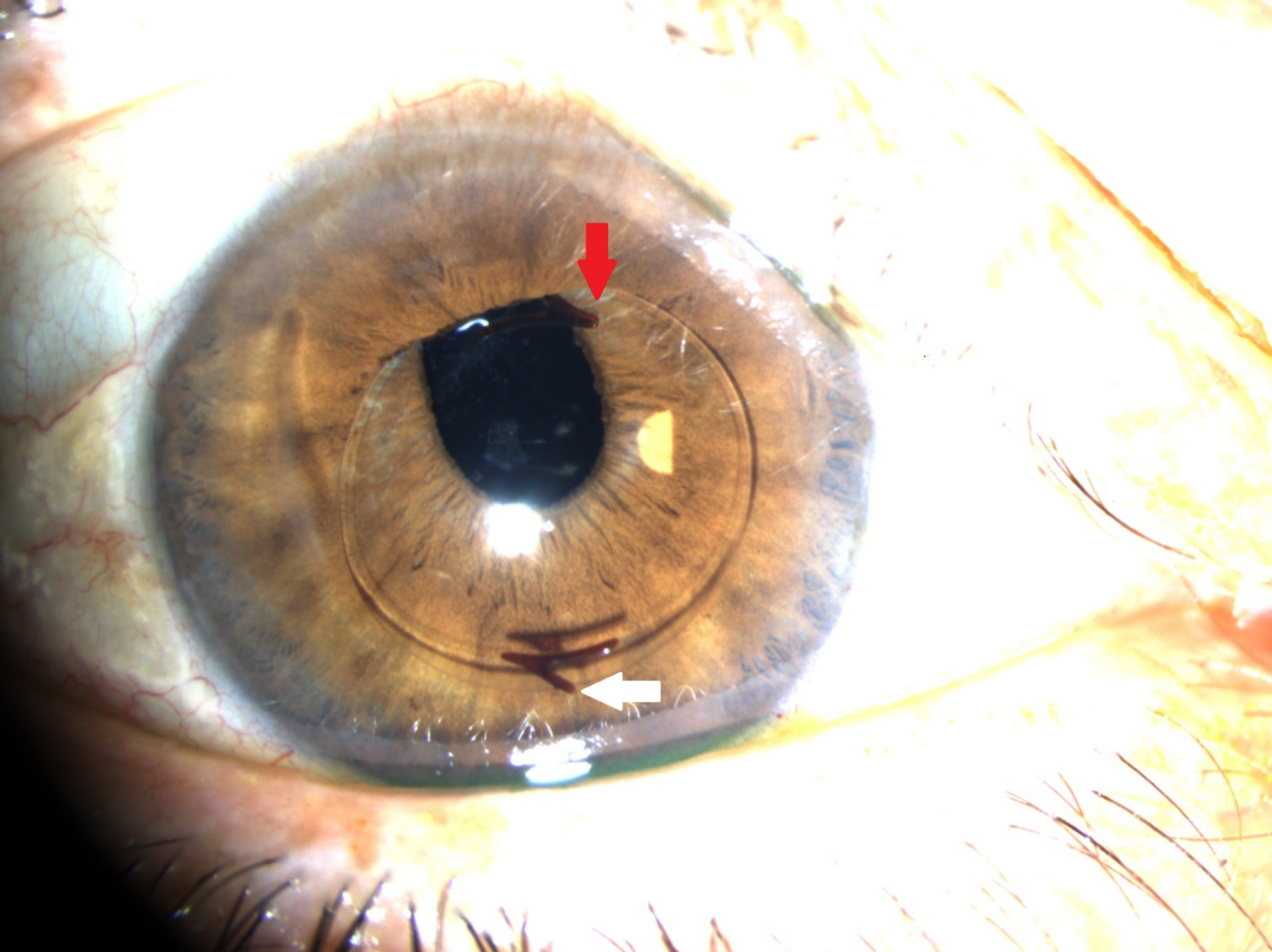
Iris optic capture demonstrating anterior dislocation of the intraocular lens (red arrow) and a fractured inferior lens haptic (white arrow) in the right eye

On examination, the patient was noted to have an IOL dislocated into the anterior chamber of the right eye with inferior iris capture of the optic and a fractured inferior lens haptic as noted in Figure 1.

Other findings included mild non-proliferative diabetic retinopathy in both eyes without macular edema. There was no evidence of pupillary block, intraocular inflammation, iris chafing, or vitreous prolapse. The patient’s visual acuity was 20/20 and the intraocular pressure was within normal limits. Given the lack of ocular symptoms and his unstable cardiac condition, observation with close follow up was recommended. The patient remained asymptomatic until five months later when he developed photophobia, hyperemia, and was found on examination to have intraocular inflammation with associated macular edema. Visual acuity in the eye at this time was 20/100. The implant's position had not changed since the previous visit. Due to his unstable cardiac status, surgical options were limited, and the patient was treated with three rounds of subtenon's triamcinolone acetonide (40mg) injections at six- to eight-week intervals along with topical prednisolone acetate 1% drops. Following cardiac clearance, the dislocated IOL was surgically explanted and the patient was left aphakic. The explanted lens was noted to be a three-piece flexible silicone lens with polyimide haptics. In the months following, the patient was noted to have complete resolution of intraocular inflammation and macular edema. His final best-corrected visual acuity was 20/20.

## Discussion

Although cataract surgery with IOL implantation has a very high success rate, intraocular lens dislocation may rarely occur postoperatively. The frequency of IOL dislocation ranges from 0.2%-1.8% [4]. Early IOL dislocations are mostly due to trauma and/or extreme Valsalva maneuvers. In a retrospective case series of 86 consecutive cases of late explanted IOLs, the IOL dislocated on average 8.5 years after surgery [5]. Late IOL dislocation resulted from zonular weakness with the associated conditions: pseudoexfoliation, prior vitreoretinal surgery, and a history of trauma or uveitis [3]. Our patient did not have a history of any of these conditions. With IOL dislocation, most patients present with pain, redness, and visual changes. Interestingly, our patient was asymptomatic at diagnosis ten months after the presumed causative event of chest compressions and remained asymptomatic until five months post-diagnosis.

One of the causative factors for haptic breakage in our case was the polyimide haptic material. Stallings and associates report two cases of explanted IOL where the polyimide haptics shattered into multiple pieces, one after removal and the other with spontaneous in situ haptic breakage [4]. Valsalva maneuvers may contribute to haptic instability and breakage in such cases. Kim et al. describe a case of spontaneous fracture of an implanted posterior chamber polyimide IOL haptic in a patient who gave a history of straining [5]. Given that our patient’s history lacks orbital trauma or other known predisposing conditions for IOL dislocation, chest compressions were the likely causative event whereby increased thoracic pressure, and a resultant secondary rise in venous blood pressure, was transmitted to the eye; an acute increase in intraocular pressure, resulting in a sudden pressure gradient in the eye, led to haptic breakage.

Polyimide haptics were first patented in 1988 and have been widely used since its introduction. Polyimide used in the aviation industry to insulate electrical wiring has been shown to become brittle over time in warm and moist environments [6]. The same mechanism of failure is prone to occur in the human eye. With the improvement in surgical techniques and the low risks of IOL implantation, more cases are being done yearly around the world. Many of these implants are in individuals in their 5th or 6th decade of life, and with the longevity of the population increasing, it is anticipated treating physicians will be increasingly presented with complications of polyimide haptic failure.

## Conclusions

Polyimide haptic failure and lens dislocation are late complications of cataract surgery. Polyimide haptics have been widely used since their introduction in 1988. Polyimide is known to become brittle over time in warm and moist environments, like the human eye. Valsalva maneuvers may contribute to haptic instability and breakage. With the improvement in surgical techniques and the low risks of IOL implantation, an ever-increasing number of cataract surgical cases are being done yearly around the world. Many of these implants are in individuals in their 5th or 6th decade of life, and with the longevity of the population increasing, physicians will be increasingly presented with complications of polyimide haptic failure. Ophthalmologists and optometrists should be aware of its possibility after cardiac compressions in at-risk patients.
